# Supplementary figures and images for: Profiling the baseline performance and limits of machine learning models for adaptive immune receptor repertoire classification
Source: Gigascience. 2022 May 25;11:giac046. doi: 10.1093/gigascience/giac046 (PMC9154052; doi:10.1093/gigascience/giac046)

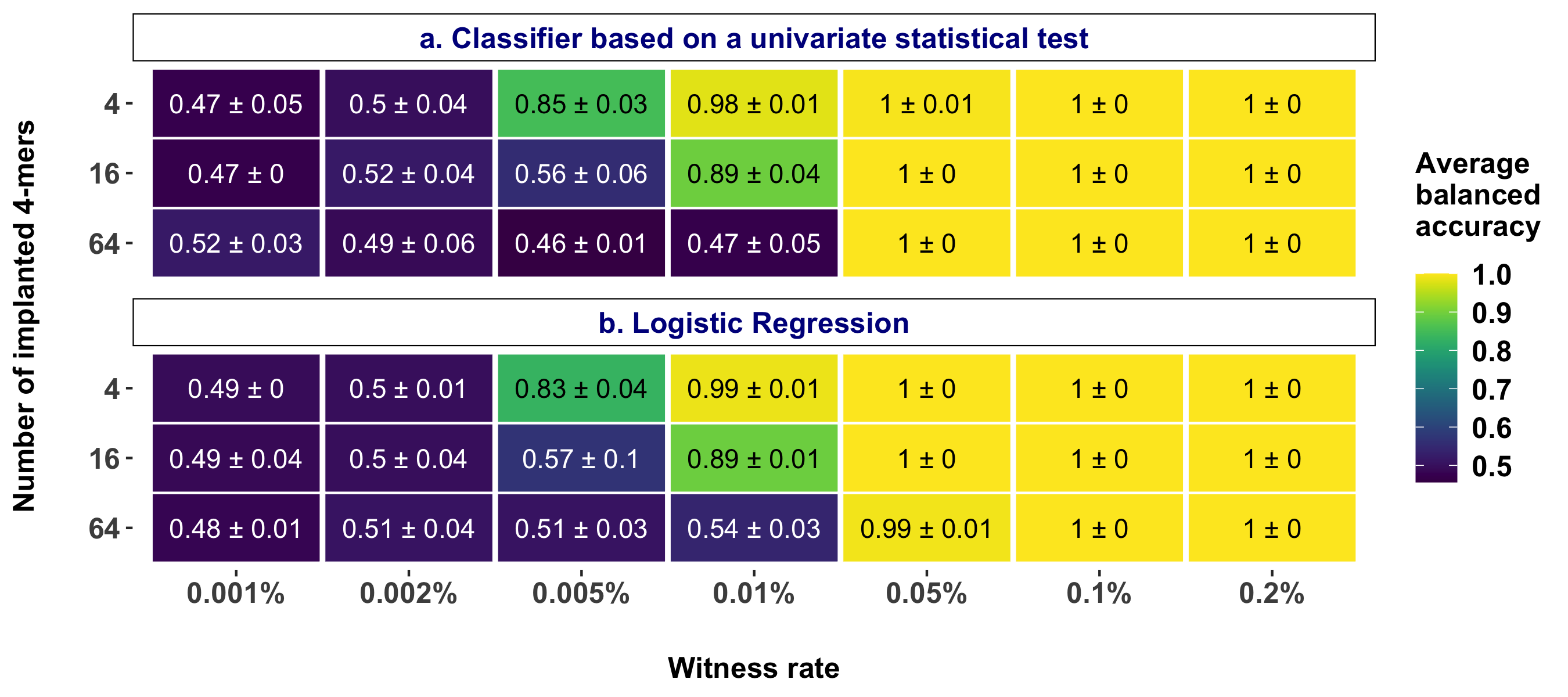

Supplement: giac046_Supplemental_Files [file giac046_supplemental_files.zip › Figure_S11_Supplementary Material.png]

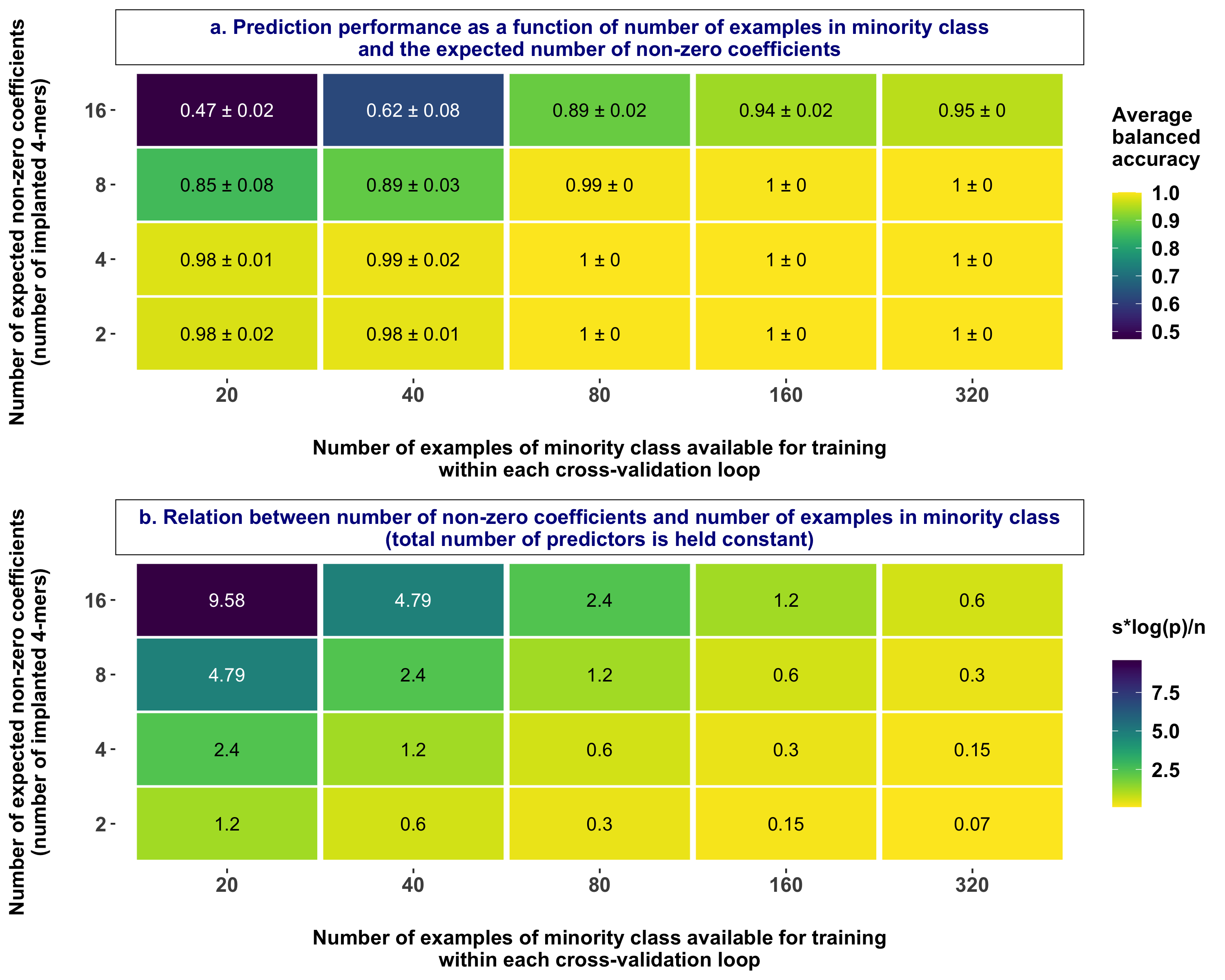

Supplement: giac046_Supplemental_Files [file giac046_supplemental_files.zip › Figure_S12_Supplementary Material.png]

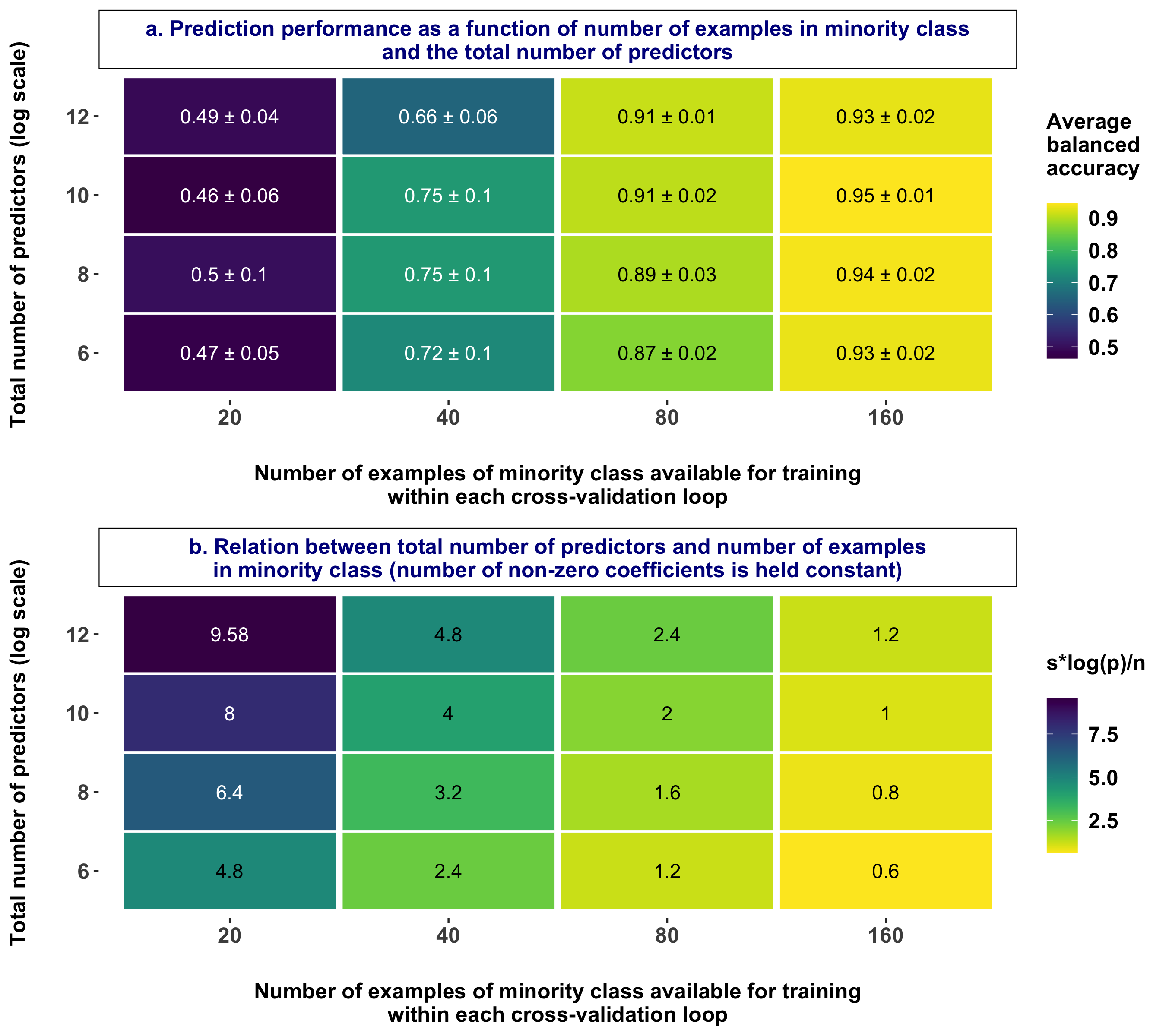

Supplement: giac046_Supplemental_Files [file giac046_supplemental_files.zip › Figure_S13_Supplementary Material.png]

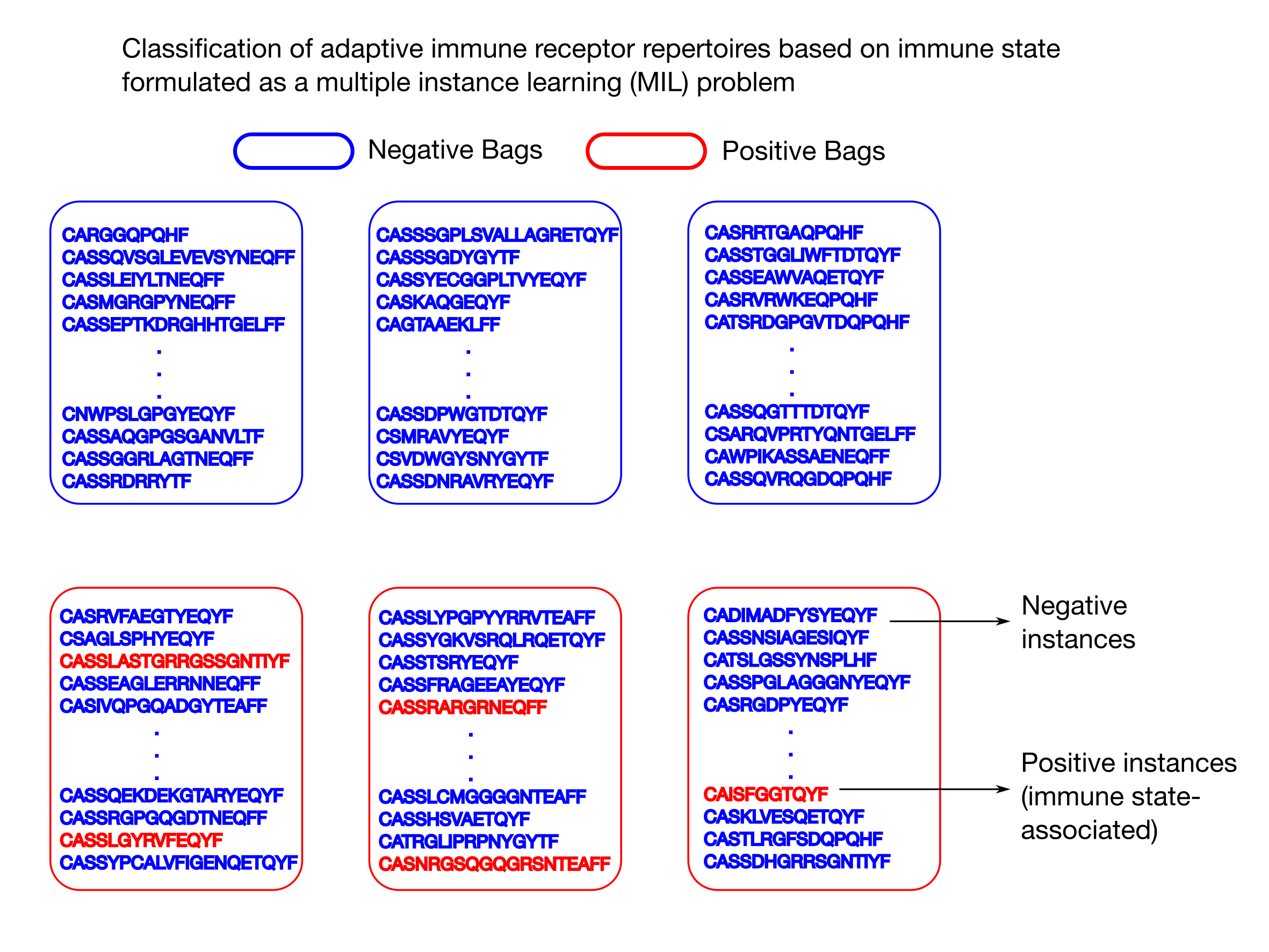

Supplement: giac046_Supplemental_Files [file giac046_supplemental_files.zip › Figure_S1_Supplementary Material.png]

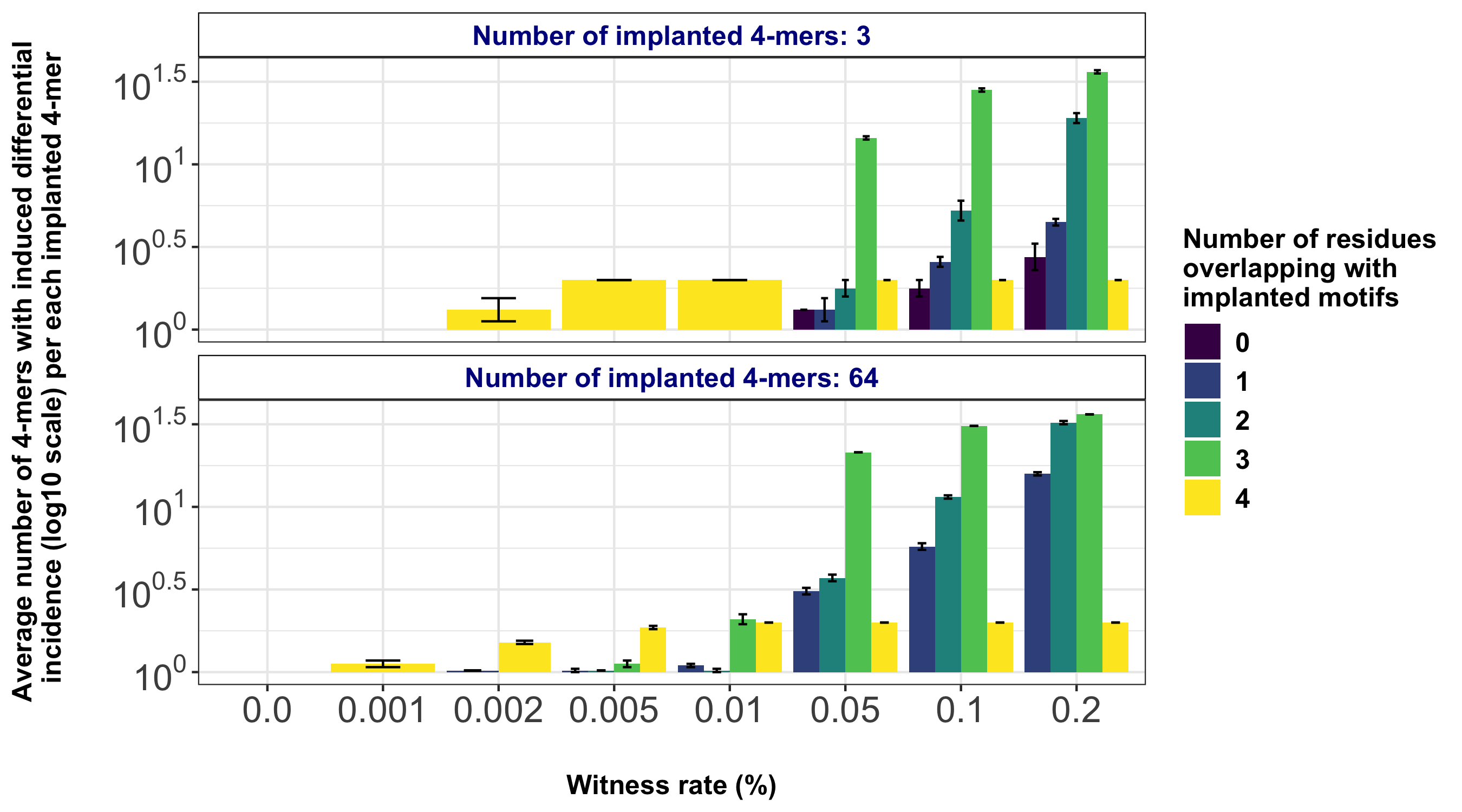

Supplement: giac046_Supplemental_Files [file giac046_supplemental_files.zip › Figure_S2_Supplementary Material.png]

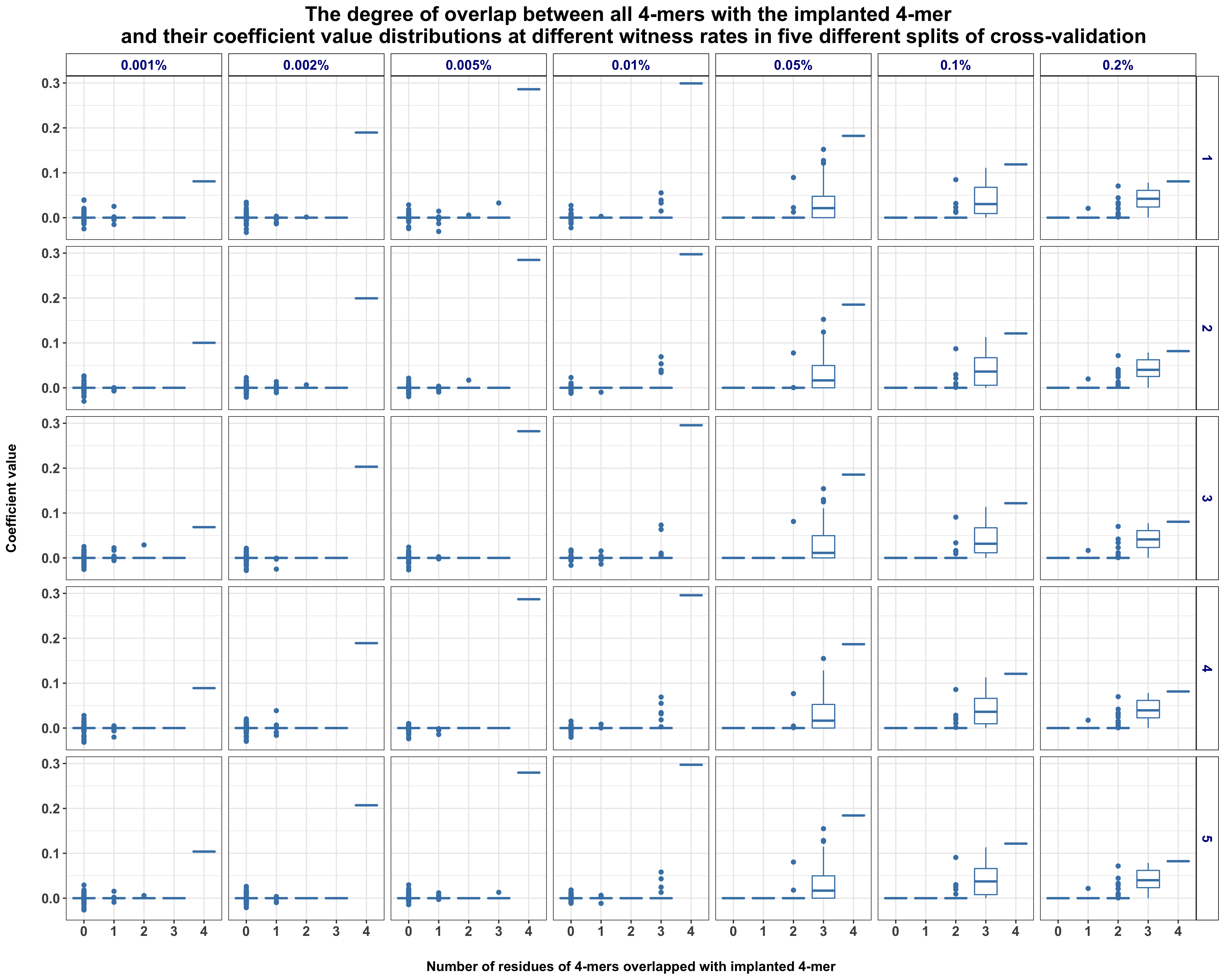

Supplement: giac046_Supplemental_Files [file giac046_supplemental_files.zip › Figure_S3_Supplementary Material.png]

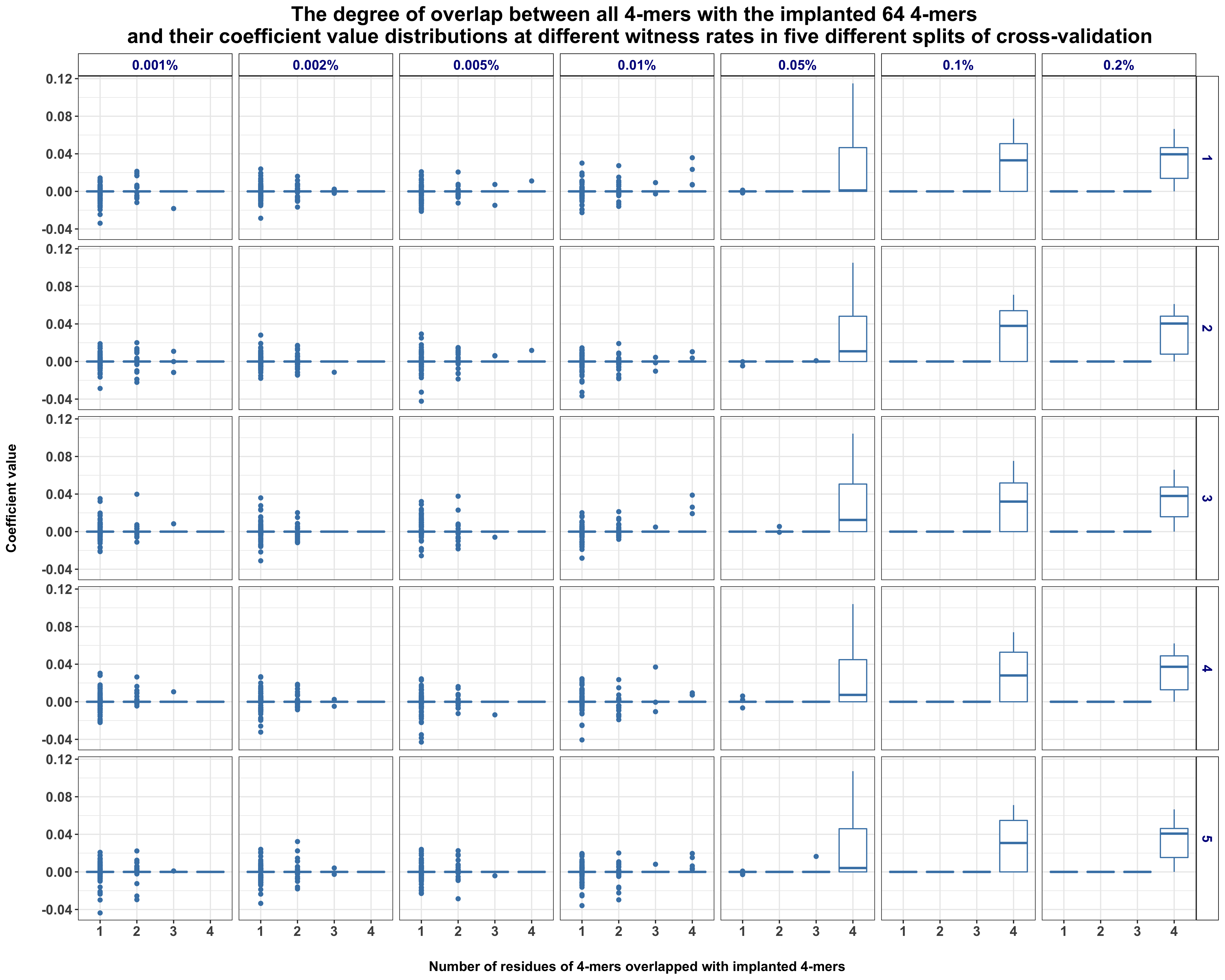

Supplement: giac046_Supplemental_Files [file giac046_supplemental_files.zip › Figure_S4_Supplementary Material.png]

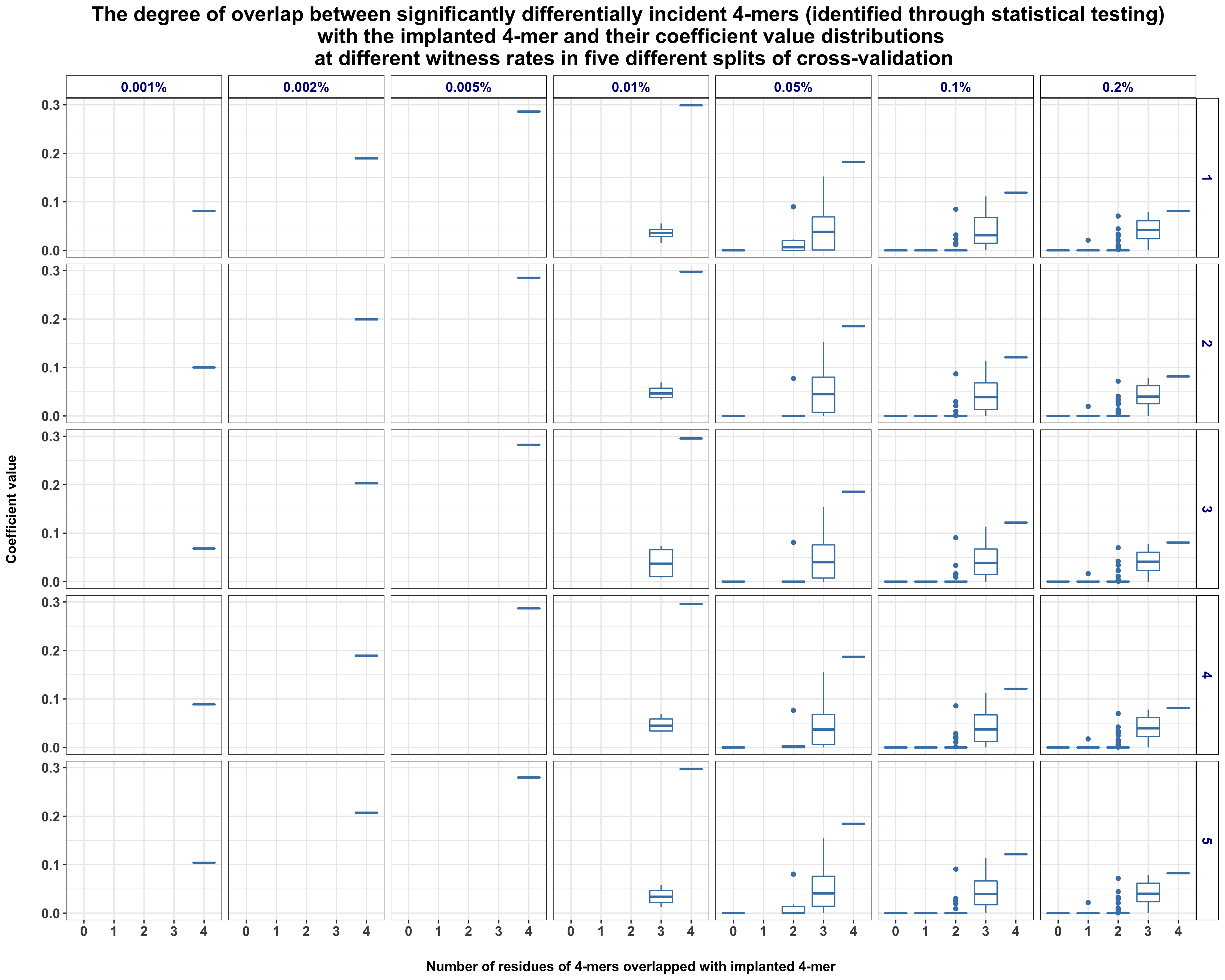

Supplement: giac046_Supplemental_Files [file giac046_supplemental_files.zip › Figure_S5_Supplementary Material.png]

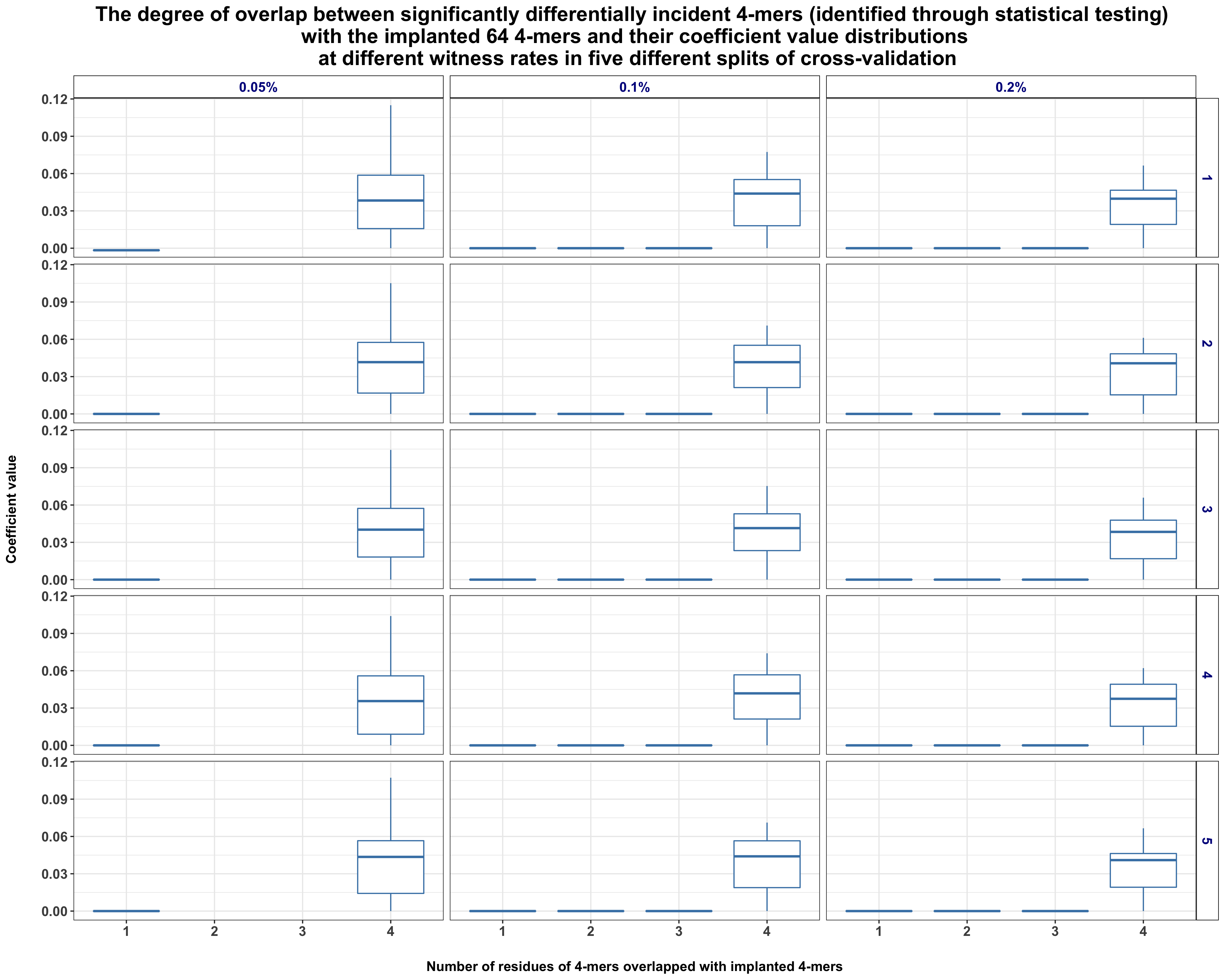

Supplement: giac046_Supplemental_Files [file giac046_supplemental_files.zip › Figure_S6_Supplementary Material.png]

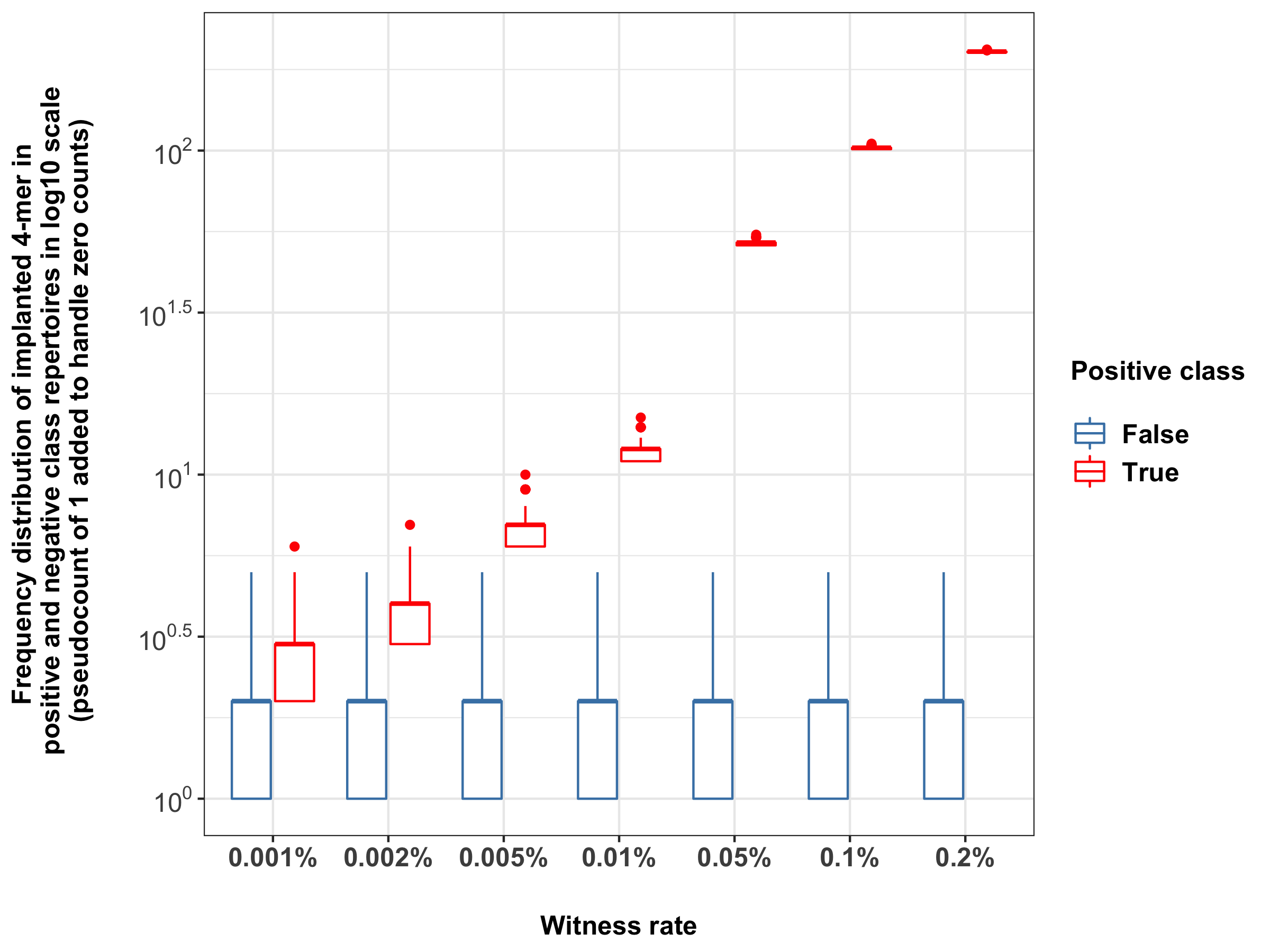

Supplement: giac046_Supplemental_Files [file giac046_supplemental_files.zip › Figure_S7_Supplementary Material.png]

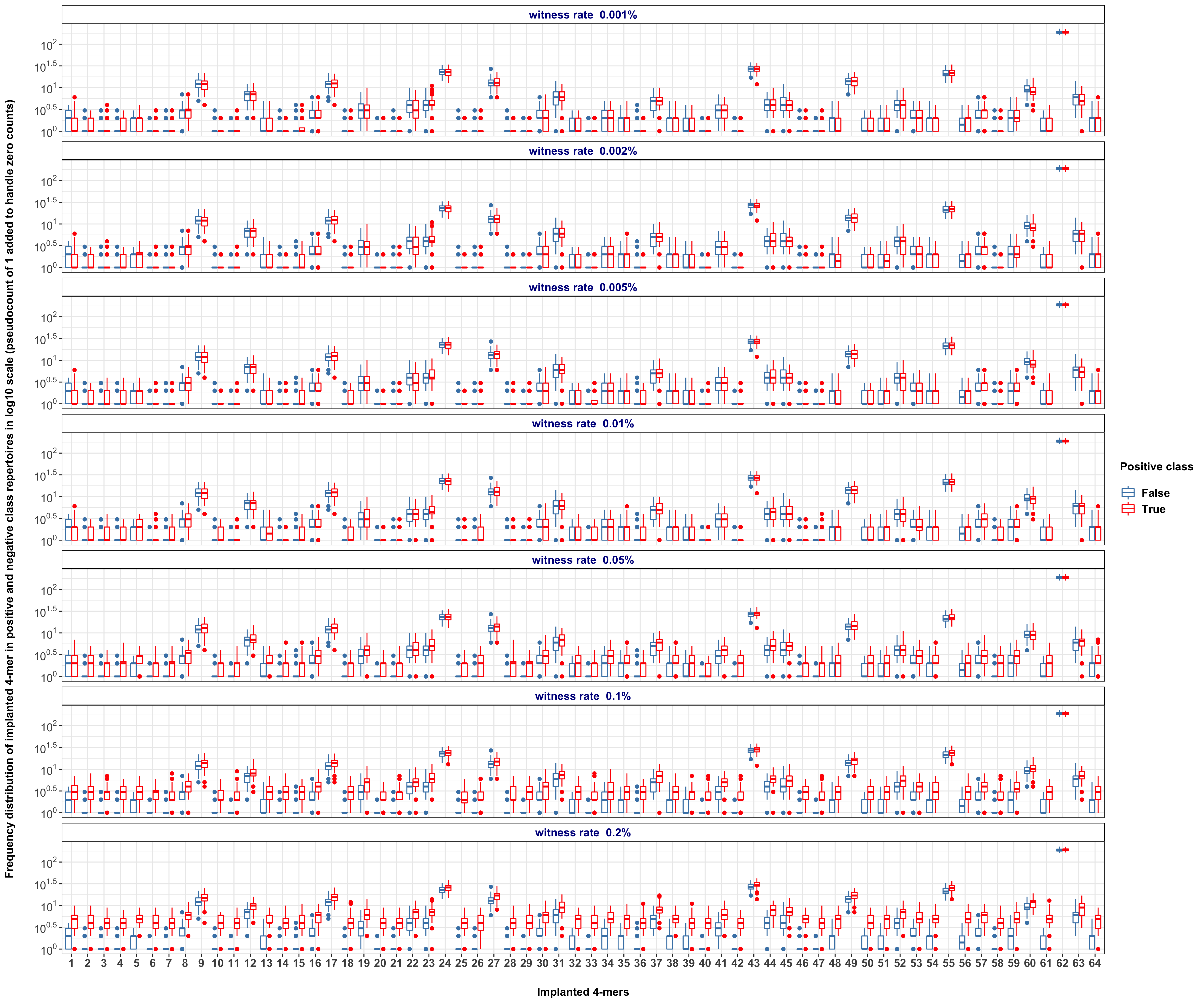

Supplement: giac046_Supplemental_Files [file giac046_supplemental_files.zip › Figure_S8_Supplementary Material.png]

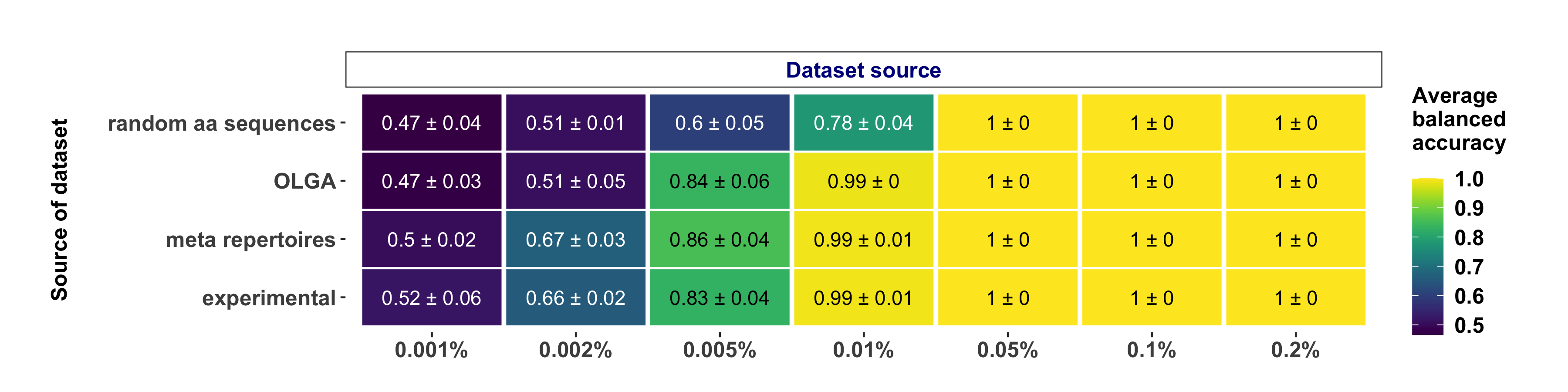

Supplement: giac046_Supplemental_Files [file giac046_supplemental_files.zip › Figure_S9_Supplementary Material.png]
