# Supplementary material for: Profiling the baseline performance and limits of machine learning models for adaptive immune receptor repertoire classification
Source: Gigascience. 2022 May 25;11:giac046. doi: 10.1093/gigascience/giac046 (PMC9154052; doi:10.1093/gigascience/giac046)
Supplement: giac046_Response_to_Reviewer_Comments_Original_Submission [file giac046_response_to_reviewer_comments_original_submission.pdf]

Author's Response To Reviewer Comments

Close

We thank all the reviewers for their constructive suggestions that allowed us to improve the manuscript. Briefly, the suggestions of the reviewers allowed us to (a) emphasise the nature of high sparsity of data in this application domain, (b) demonstrate that suited sparse models including feature selection-aided models perform adequately given sufficient statistical power, (c) relate to theoretical results of sparse model theory, (d) clarify on confusing aspects of performance metrics, (e) illuminate the readers on formulating the problem setup in terms of multiple instance learning and (f) improve consistency of reporting including language. Below, we respond to each comment, where the text of our responses is in black font. In the revised manuscript file, all the changes are shown in blue font. Where possible, the changes made in the main manuscript are also presented in this response document for corresponding comments of the reviewers.

Reviewer reports:

Reviewer #1: The manuscript by Kanduri et al benchmarks baseline machine-learning method on simulated sequencing data of adaptive immune receptors to predict immune states of individuals by detecting antigen-specific signatures. Given that there is a volume of publication using a wide variety of different machine learning techniques with the promise of clinical diagnostics on such data, the goal of the study is to set baseline expectations. From an application standpoint, I believe that the study motivated and useful to the communitee. From a signal processing standpoint, many aspects of the study are trivial consequences of the simulation choices: sparse estimators are good for prediction when the signal is generated from sparse coefficients. Though I do not know well this application community, it seems to me that the manuscript is valuable because it casts this knowledge in a specific application setting, however it should discuss a bit more the fundamental statistical
reasons that underly the empirical findings. I give below some major and minor comment to help make the study more solid.

We thank the reviewer for the overall positive assessment of the manuscript and for the suggestions to make the study even more solid. We performed several new experiments based on the suggestions and agree that the added findings will be useful to the scientific community within this application domain.

1. Plausibility of the simulations

The validity of the findings relies crucial on the simulations, in particular the hypotheses of extreme sparsity. These hypotheses need to be discussed more in details, with references to back them. The amount of sparsity as detailed in table 1, is huge, which strongly favors sparse models.

We agree that the validity of the findings relies on the assumptions underlying the simulations. The patterns associated with a positive class in this particular application domain can be as rare as one positive feature amongst one million features. We added these details and references to back them in the introduction section on page 2.

On page 2: "One of the main challenges for AIRR-ML methodology is that the patterns associated with an immune state (positive instances in MIL terminology) can be as rare as one antigen-binding AIR per million lymphocytes [19,36]. A similarly low incidence of positive instances was also reported in previous studies that used ML for classification of immune states based on AIRR data (e.g., [23,26]). However, notably, the reported degree of rareness of identified positive instances can vary between immune states as well as based on the sampling depth and statistical power of the studies."

2. Another baseline, natural given the sparsity

I do realize that the goal of this study is not do an exhaustive comparison of all machine learning methods --an impossible task--, however for someone knowledgeable about sparse signal processing, In particular, the study begs the question of whether univariate tests on appropriate k-mer can be enough,

an alley suggested by the authors on page 7. This option should be studied empirical, as it would provide important practical methods.

We thank the Reviewer for this very interesting question. To address this question, we built a classifier that uses a univariate statistical test for feature selection and compared its performance to the standard lasso method used throughout this study. The results of these new analyses are presented on page 15, where other ML methods like RF and SVC were compared. We described the new classifier under methods on page 22. The supplementary figure (Figure S11) that shows the results of these comparisons is shown on page 40.

On page 15: "Following the observation that sparse models based on L1-penalization suited this particular problem setup, a natural curiosity would be whether a feature selection step combined with a non-penalized model would exhibit similar performance levels as penalized counterparts. To this end, we built a feature selection-aided classifier and compared its performance to L1-penalized logistic regression. Expectedly, as also observed in the analyses of Figure S5 and Figure S6 described above, both the models exhibited similar performance across all the scenarios tested (Figure S11). "
On page 22: "In addition to the off-the-shelf ML methods, we built a feature selection-aided classifier as another baseline to compare with the traditional sparse models during the revision process. Briefly, we used a student's t-test to compare the two class frequency distributions for each k-mer (as many tests as k-mers) and used the p-value of the resulting tests as a hyperparameter that was optimized for in the inner-loop of a nested cross-validation. Only the features selected based on the optimal hyperparameter were included in the design matrix on which a traditional logistic regression with no penalization was fit. The method was implemented inside immuneML [61] and a separate docker image running the particular feature branch is provided under the code availability section."
On page 39:

Figure S11. Comparison of the performance of a feature selection-aided classifier with that of an L1-penalized logistic regression. Performance estimates of two different models in a binary classification of a balanced, labelled AIRR dataset where the signal in positive class examples was composed of varying numbers of 4-mers (on the y-axis) and are known at the explored witness rates (x-axis). (a) Performance estimates of a classifier that was aided by a feature selection step based on a univariate statistical test. (b) Performance estimates of an L1-penalized logistic regression model. The mean balanced accuracy of 5-fold nested cross-validation was computed in three independent replications. The colour coding shows the mean and standard deviation of the performance estimate obtained by three independent replications.

3. Link to sparse model theory

A vast variety of theoretical results state that a sparse model will be successful for n proportional to s log(p) where n here would be the number of samples in the minority class, s would be the number of non-zero coefficients.

A good summary of these results can be found in the book "Statistical learning with sparsity: the lasso and generalizations T Hastie, R Tibshirani, M Wainwright - 2019"

It would be interesting to see how these theoretical scaling match results, for instance those on figure 3.

Thanks for the tips and references. The analyses of the manuscript in its previous form (Figure 3) were not best designed to make good comparisons with the suggested theoretical results because none of the simulations varied both the number of examples of minority class, n, and the expected number of non-zero coefficients, s, simultaneously, whereas the total number of predictors in log scale, log(p) was held constant in most of the simulation scenarios. To address this suggestion, we designed two new experiments and presented the findings in the supplementary information section on page 40. Two new supplementary figures (Figure S12 and Figure S13) were added on pages 41 and 42. We believe the theoretical results will be useful during study design in this particular application domain characterised by high dimensionality and sparse signals and we discussed it in the discussion section on page 16.

On page 16: "Third, we observed that the prediction performance of an L1-penalized logistic regression, at a constant witness rate of 0.01%, improved with increasing n proportional to s*log(p), where n refers to the number of examples of the minority class (the class that has few examples in an imbalanced binary classification problem), p refers to the total number of predictors (4-mers), and s refers to the number of non-zero coefficients of ML model (Supplementary text and Figure S12 and Figure S13). This

observation is in agreement with the theoretical results of sparse model theory [50], which may serve the study design of AIRR studies that are characterized by high dimensionality and sparse signals. Notably, however, the effect sizes of coefficients and the sum of non-zero coefficients which can vary with witness rate, sequencing depth and between different immune states may also have an impact on the success of suited sparse models at different sample sizes and are warranted to be investigated in future studies."

On page 40: "Relating the empirical sample complexity with sparse model theory
The patterns associated with an immune state can be as rare as one antigen-binding AIR per million lymphocytes and were found to occur at a low incidence in previous studies [19,23,26,36]. Sparse models tested in this study are thus particularly suited to the problem setup. In Figure 3.a, by considering the prediction performance as a function of sample size and witness rate in balanced datasets, we empirically assessed the sample complexity while holding the total number of predictors (4-mers) and the expected number of non-zero coefficients of the ML model (number of implanted 4-mers) constant. The theoretical results of sparse model theory [50] state that a sparse model will be successful for n proportional to s*log(p), where n refers to the number of examples of the minority class (here minority class refer to the class that has few examples in an imbalanced binary classification problem), p refers to the total number of predictors, and s refers to the number of non-zero coefficients. We set out to understand if the sample complexity of sparse models used in this study match with the theoretical results. Specifically, we assessed the prediction performance of the L1-penalized logistic regression as a function of the number of examples in minority class with (i) a varying number of expected non-zero coefficients while holding the number of predictors constant (Figure S12) and (ii) a varying number of predictors while holding the expected number of non-zero coefficients constant (Figure S13). In agreement with the theoretical results, we observed that the higher the expected number of non-zero coefficients relative to the number of examples in the minority class, the lower the prediction performance (Figure S12). Similarly, we observed a qualitative distinction in performance levels between high and low s*log(p) when holding s constant (Figure S13). However, we observed no differences in performance with a decreasing number of total predictors when the number of examples in the minority class was constant. "

On page 41:

Figure S12. Performance as a function of the number of examples in minority class and the expected number of non-zero coefficients. Performance estimates of an L1-penalized logistic regression in a binary classification of a balanced, labelled AIRR dataset where the signal in positive class examples was composed of varying numbers of 4-mers (on the y-axis) and are known at a witness rate of 0.01%, and the number of examples of minority class available for training within each cross-validation loop is varied (on the x-axis). (a) Performance estimates of an L1-penalized logistic regression model. The mean balanced accuracy of 5-fold nested cross-validation was computed in three independent replications. The colour coding shows the mean and standard deviation of the performance estimate obtained by three independent replications. (b) Relation between the expected number of non-zero coefficients, s (y-axis), and the number of examples in minority class, n (x-axis), and number of predictors in log scale, log(p), which is held constant as 12. For each cell of the heatmap in panel (a), the corresponding cell in panel (b) shows the ratio s*log(p)/n and the colour coding represents the range of this ratio. The figure shows that the low prediction performance on panel (a) correlates negatively with a high ratio of s*log(p)/n.

On page 42:

Figure S13. Performance as a function of the number of examples in the minority class and the total number of predictors. Performance estimates of an L1-penalized logistic regression in a binary classification of a balanced, labelled AIRR dataset where the signal in positive class examples was composed of sixteen 4-mers at a witness rate of 0.01% that was held constant, while the number of examples of minority class available for training within each cross-validation loop is varied (on the x-axis) and the total number of predictors was varied on the x-axis. Notably, the varying total number of predictors on the x-axis always included the predictors that constitute the signal. (a) Performance estimates of an L1-penalized logistic regression model. The mean balanced accuracy of 5-fold nested cross-validation was computed in three independent replications. The colour coding shows the mean and standard deviation of the performance estimate obtained by three independent replications. (b) Relation between the expected number of non-zero coefficients, s, and the number of examples in minority class, n, and number of predictors in log scale, log(p). For each cell of the heatmap in panel (a), the corresponding cell in panel (b) shows the ratio s*log(p)/n and the colour coding represents the range of

this ratio.

4. Accuracy and class imbalance

It seems to me that in parts of the manuscript (fig 4.a for instance) accuracy is compared across different scenarios with varying class imbalance. However, accuracy is not comparable when class imbalance varies: for instance with 90% positive class, a classifier that always choose the positive label will have .9 accuracy. In this light, I don't understand fig 4.a, in which even for large class imbalance accuracy goes to .5. In addition, the typical good practice is to use a metric for which decision under chance are not affected by class imbalance, such as area under the curve of the ROC curve.

We agree with the Reviewer that the color legends of the figures were confusing. In the previous version of the manuscript, we stated only in the figure legends that we used "balanced accuracy" (https://scikit-learn.org/stable/modules/generated/sklearn.metrics.balanced_accuracy_score.html), whereas the color legends on the figures just mentioned accuracy. We have now modified the color legends of all heatmaps to clarify that we used a balanced accuracy
(pages 8, 9, 11, 13, 21, 38-42). We also added this to the Methods section on page 23.

On page 23: "We replicated each investigation three times on separate datasets and the average performance metric of the 3 replications together with variation is reported. The reported performance metric of each replication is the balanced accuracy [63] obtained through nested CV."

5. Comparison with SVC

The manuscript mentions that a Support Vector Classifier is also benchmarked, however it does not give details on which specific SVC is used. A crucial point is the kernel used: with a linear kernel, the SVC is a linear model, while with another kernel (RBF kernel, for instance), the SVC is a much more complex model and is not expected to behave well in large p, small n problems. Also, I suspect that the SVC is used with the l2 regularization. A linear SVC with l1 regularization would likely have similar performance as the l1-penalized logistic regression, as it is a model of the same nature. These details should be added; ideally, if the model benchmarked is not a linear SVC, a linear SVC should be benchmarked, to give a baseline (though the default l2 regularization can be used, to stick to common practices).

We have now replaced the previous SVC results with new results on page 39 based on a SVC that uses a linear kernel and l1-penalization. We modified the relevant results section on page 15 to reflect the findings of similar behavior of l1-penalized logistic regression and l1-penalized linear kernel-based SVC. We added details of kernel choice and regularization to the Methods section on page 22.

On page 15: "We observed that both logistic regression and SVC exhibited similar performance on this classification problem, whereas the RF performed poorly (Figure S10.a). We believe this is due to the stronger degree of regularization of the L1-penalization used by both logistic regression and SVC that suited the problem domain characterized by sparse signals. "

On page 39:

Figure S10. (Relates to Figures 2-5). Impact of the choice of ML method and the hyperparameter spaces of explored ML methods. (a) Performance estimates of different ML models (on y-axis) in a binary classification of balanced, labeled AIRR datasets, where the signal in positive class examples composed of 4-mers are known at the explored witness rates (on x-axis). The mean balanced accuracy of a 5-fold cross-validation was computed in three independent replications. The color coding shows the mean and standard deviation of the performance estimate obtained by three independent replications.

6. Wording in the conclusion

The conclusion starts with "To help the scientific community in avoiding futile efforts of developing...". The word futile is too strong and the phrasing will not encourage healthy scientific discussion.

The sentence has been rephrased accordingly on page 19.

On page 19: "To help the scientific community in reorienting efforts of developing novel ML methodology

for those scenarios of AIRR classification where baseline methods fail, we profiled the baseline performance of AIRR ML models across a diverse set of challenges with increasing levels of complexities for the classification problem."

I try to sign my reviews as much as possible.

Gaël Varoquaux

Reviewer #2: General opinion: approved with minor changes

Comments: The manuscript profiles machine learning methods for AIRR T-cell receptor dataset immune state label prediction to establish the baseline performance of such methods across a diverse set of challenges. Simulated datasets with variable properties are used to provide a large amount of benchmarking datasets with known immune state signals while reflecting the natural complexity of experimental datasets. Their results provide insights on the current limits posed by basic dataset properties to baseline ML models and establish a frontier of improvement of AIRR ML research.

The manuscript is understandable and well structured in the approach to comparisons as well as solid conclusions. The graphics are clear and consistent and support the manuscript. Very interesting insight into the importance of single individual variable parameters such as sample size or witness rate on the general accuracy.

The advantage of the results to the scientific community is that it offers an evaluation of classical ML methods, provides large and specialized AIRR benchmark datasets, and allows further development and benchmarking of more sophisticated ML methods.

The manuscript is overall well-written and we endorse it with minor changes:

Thanks for the overall positive assessment of the manuscript and for all the suggestions on minor changes. We have corrected all the errors you noticed.

In paragraph Impact of noise on classification performance (page 14) the sentence "but enriched above a baseline in positive class examples" should be corrected with "but being enriched above a baseline in positive class examples"

Corrected on page 14.

In paragraph Machine learning models (methods section, page 21) "lasso" should be corrected with "Lasso".

Corrected, now on page 22 in revised version.

In paragraph Machine learning models (methods section, page 21) " '- ' " should be corrected with "'-'" and "jdenoting» with "j denoting».

Corrected, now on page 22 in revised version.

In the discussion the sentence "which aligns with the observations that that the majority of the possible contacts between TCR and peptide" should be corrected with "which aligns with the observations that the majority of the possible contacts between TCR and peptide"

Corrected, now on page 15 in revised version.

Keep comparisons like size>500 and size > 500 concise
Check for missing whitespace as in the description of the figure 1(b): …(5 x 105 % of sequence..
Same in cases like ≈90% | ≈ 90 % or n=60 | n = 60

Corrected on all pages where mathematical symbols are used.

Reviewer #3: The article "Profiling the baseline performance and limits of machine learning models for

adaptive immune receptor repertoire classification by
Kanduri1 et al. describes the construction of suitable reference benchmarks data-sets to guide new AIRR ML classification methods.

The article is interesting and potentially useful in defining benchmark data sets and criteria for constructing specialized AIRR benchmark datasets for the community of researcher interested in AIRR.

The authors following previous indications about model reproducibility and availability also provide a docker container which include all data and procedures to reproduce the study.

The article is sufficiently well written although at time a bit full of details which perhaps could be synthesised further (this has already been done in pictures and tables).

I don't have major concerns. Only a couple of notes.

Thanks for the overall positive assessment of the manuscript and the suggestion of including an illustration on multiple instance learning formulation of the AIRR-ML problem. We included the figure and explained the reason behind some of our choices below.

Would be good to have a figure or diagram showing an example of bags containing receptors and associated witnesses. It could illuminate the reader not familiar with Multiple instanvd learning.

We have now referred to a newly added supplementary figure (Figure S1 on page 31) in the introduction section on page 2, where AIRR-ML formulation as a multiple instance learning problem has been introduced.

On page 31:

Figure S1. An illustration of formulating classification of immune states based on AIRR data as a multiple instance learning problem. Some of the published methods [23,28,32] aptly considered AIRR classification as a multiple instance learning problem (MIL) [35] consisting of repertoires as bags, receptors as instances, and immune state-associated receptors as witnesses. Here, the witnesses (positive instances) are shown in red text, whereas the blue text represents negative instances. Notably, the positive instances can be as rare as one in a million instances. The MIL methods use different pooling functions to learn the relationship between the labels of instances of a bag and the label of a bag (color of bags here).

Would be good to have line commands for the generation of data sets (in the case, for instance, of use of Olga). I understand these are inside the docker container but the reader that is not interested in the whole container might find useful to have access to pieces of the pipeline so to use this or that tool (being it in immuneML, in Olga, etc.).

The line commands, in the form of shell scripts, to generate the AIRR datasets are provided in this link: https://doi.org/10.11582/2021.00038. The 20 MB archive when downloaded contains a README file that describes these shell scripts under the section titled "Generation of raw AIRR datasets". The URL is made available under the Data and Source Code availability section on page 24.

Curiosity: why have the authors used Olga and not the mate Igor?

OLGA directly generates amino acid receptor sequences that are used in this study, whereas one would need to have an additional step of translation of nucleotide sequences to amino acid sequences in the case of IgoR.

Why is the performance metric in model training the accuracy and not, for instance, the F1-score? Any particular reason?

We have in the revised version clarified that what is reported throughout the manuscript is "balanced accuracy" (arithmetic mean of sensitivity and specificity). We realized that was not clear in the previous version (we have now added this to the color legends of all the figures to make this clearer, see also response to comment #4 of reviewer #1). We originally considered alternative metrics, like F1 score or AUROC. Our main motivation for choosing balanced accuracy was the ease of interpretability - for

balanced datasets (the majority of datasets in the study) the balanced accuracy becomes plain accuracy, which has the simple interpretation as proportion of correctly classified repertoires. For imbalanced datasets it still has a relatively straightforward interpretation (as the mean of sensitivity and specificity), while avoiding the easily misleading behavior of plain accuracy on such settings (where high values for plain accuracy can be achieved by almost always predicting the majority class, if classes are severely imbalanced) .

Close
