# Supplementary material for: Profiling the baseline performance and limits of machine learning models for adaptive immune receptor repertoire classification
Source: Gigascience. 2022 May 25;11:giac046. doi: 10.1093/gigascience/giac046 (PMC9154052; doi:10.1093/gigascience/giac046)
Supplement: giac046_Response_to_Reviewer_Comments_Revision_1 [file giac046_response_to_reviewer_comments_revision_1.pdf]

Author's Response To Reviewer Comments

Close

We thank the reviewer for the constructive suggestions that allowed us to further improve the manuscript. We also agree with the reviewer's opinion that with all these changes the manuscript is pretty much complete and readable. In this minor revision round, we agreed with all the suggestions of the reviewer and made the suggested changes. Below, we respond to each of the 3 comments, where the text of our responses is in black font. In the revised manuscript file, all the changes are shown in blue font. Where possible, the changes made in the main manuscript are also presented in this response document for corresponding comments of the reviewer.

Reviewer #1: I would like to thank the authors for the work done on their manuscript, in particular adding the experiments that enable linking to sparse-recovery theory. In my opinion, the manuscript brings a lot of value to the application community and is pretty much complete. A few details come to my mind that could help its message be most accurate.

Because of my suggestions, the authors have used an l1 penalty in the SVC. This worked well in terms of prediction. However, it is not the default. I think that the authors should stress this and be precise on the penalty each time they mention the SVC. In addition, I think that there would be value in performing an additional experiment with an l2 penality (which is the default) to stress the importance of the l1 penalty. The message should stress that the penality (l1 vs l2) is importance, but less the loss (log reg vs SVC).

We agree with the reviewer that mentioning the type of penalty used when referring to SVC is important to emphasize that it is penalization rather than the loss that is important for predictive performance for this problem setting. In the revised version, we mentioned the type of penalty used whenever referring to SVC. To stress the importance of L1 penalty, we added one more experiment using L2 penalty when comparing ML methods (one more row in panel (a) of Figure S10). We also emphasised this message in the main results section on page 15.

On page 15: "This highlights that a stronger degree of regularization (L1 vs L2) rather than the loss function (logistic regression vs SVC) is important for obtaining a better prediction performance for AIRR classification, given the assumption that immune state-associated patterns are rare."

On page 40: One more row was added to panel (a) of Figure S10 corresponding to an SVC model with the default L2 penalty.

As a minor detail, I would invert the color scale of one of the plot plots on figure S12, S13, to stress the parallel between the two.

We agree with the reviewer that inverting the color scale of panel (b) in both Figures S12 and S13 makes it easy to convey the correlation between the (a) and (b) panels.

On page 42-43: The color scales of panel (b) in both Figures S12 and S13 have now been inverted.

Finally, I think that it is important to stress in the conclusion that all the results build on the fact that the predictive information is sparse (maybe putting this with words more familiar to the application community).

We agree with the reviewer and added a statement in the conclusion about the suitability of sparse models given the low incidence of signal in this application domain.

On page 19: "Notably, the findings demonstrated the suitability of sparse models for AIRR classification, given the assumption that the patterns associated with an immune state occur at low frequency and can be as rare as one antigen-binding AIR per million lymphocytes."

Close
