# Supplementary material for: Profiling the baseline performance and limits of machine learning models for adaptive immune receptor repertoire classification
Source: Gigascience. 2022 May 25;11:giac046. doi: 10.1093/gigascience/giac046 (PMC9154052; doi:10.1093/gigascience/giac046)
Supplement: giac046_Response_to_Reviewer_Comments_Revision_2 [file giac046_response_to_reviewer_comments_revision_2.pdf]

Author's Response To Reviewer Comments

Close

All the editorial suggestions have been addressed.

Close
