# Supplementary material for: Profiling the baseline performance and limits of machine learning models for adaptive immune receptor repertoire classification
Source: Gigascience. 2022 May 25;11:giac046. doi: 10.1093/gigascience/giac046 (PMC9154052; doi:10.1093/gigascience/giac046)
Supplement: giac046_Reviewer_1_Report_Original_Submission — GaÃ«l Varoquaux -- 8/19/2021 Reviewed [file giac046_reviewer_1_report_original_submission.pdf]

Reviewer Report

Title: Profiling the baseline performance and limits of machine learning models for adaptive immune receptor repertoire classification

Version: Original Submission **Date:** 8/19/2021

Reviewer name: GaÃ«l Varoquaux

Reviewer Comments to Author:

The manuscript by Kanduri et al benchmarks baseline machine-learning method on simulated sequencing data of adaptive immune receptors to predict immune states of individuals by detecting antigen-specific signatures. Given that there is a volume of publication using a wide variety of different machine learning techniques with the promise of clinical diagnostics on such data, the goal of the study is to set baseline expectations. From an application standpoint, I believe that the study motivated and useful to the communitee. From a signal processing standpoint, many aspects of the study are trivial consequences of the simulation choices: sparse estimators are good for prediction when the signal is generated from sparse coefficients. Though I do not know well this application community, it seems to me that the manuscript is valuable because it casts this knowledge in a specific application setting, however it should discuss a bit more the fundamental statistical reasons that underly the empirical findings. I give below some major and minor comment to help make the study more solid.

1. Plausibility of the simulations

The validity of the findings relies crucial on the simulations, in particular the hypotheses of extreme sparsity. These hypotheses need to be discussed more in details, with references to back them. The amount of sparsity as detailed in table 1, is huge, which strongly favors sparse models.

2. Another baseline, natural given the sparsity

I do realize that the goal of this study is not do an exaustive comparison of all machine learning methods --an impossible task--, however for someone knowledgeable about sparse signal processing, In particular, the study begs the question of whether univariate tests on appropriate k-mer can be enough, an alley suggested by the authors on page 7. This option should be studied empirical, as it would provide important practical methods.

3. Link to sparse model theory

A vast variety of theoretical results state that a sparse model will be successful for n proportional to s log(p) where n here would be the number of samples in the minority class, s would be the number of non-zero coefficients.

A good summary of these results can be found in the book "Statistical learning with sparsity: the lasso and generalizations T Hastie, R Tibshirani, M Wainwright - 2019"

It would be interesting to see how these theoretical scaling match results, for instance those on figure 3.

4. Accuracy and class imbalance

It seems to me that in parts of the manuscript (fig 4.a for instance) accuracy is compared across different scenarios with varying class imbalance. However, accuracy is not comparable when class imbalance varies: for instance with 90% positive class, a classifier that always choose the positive label will have .9

accuracy. In this light, I don't understand fig 4.a, in which even for large class imbalance accuracy goes to .5. In addition, the typical good practice is to use a metric for which decision under chance are not affected by class imbalance, such as area under the curve of the ROC curve.
5. Comparison with SVC
The manuscript mentions that a Support Vector Classifier is also benchmarked, however it does not give details on which specific SVC is used. A crucial point is the kernel used: with a linear kernel, the SVC is a linear model, while with another kernel (RBF kernel, for instance), the SVC is a much more complex model and is not expected to behave well in large p, small n problems. Also, I suspect that the SVC is used with the l2 regularization. A linear SVC with l1 regularization would likely have similar performance as the l1-penalized logistic regression, as it is a model of the same nature. These details should be added; ideally, if the model benchmarked is not a linear SVC, a linear SVC should be benchmarked, to give a baseline (though the default l2 regularization can be used, to stick to common practices).
6. Wording in the conclusion
The conclusion starts with "To help the scientific community in avoiding futile efforts of developing...". The word futile is too strong and the phrasing will not encourage healthy scientific discussion.
I try to sign my reviews as much as possible.
GaÃ«l Varoquaux

Methods

Are the methods appropriate to the aims of the study, are they well described, and are necessary controls included? Choose an item.

Conclusions

Are the conclusions adequately supported by the data shown? Choose an item.

Reporting Standards

Does the manuscript adhere to the journal's guidelines on minimum standards of reporting? Choose an item.

Choose an item.

Statistics

Are you able to assess all statistics in the manuscript, including the appropriateness of statistical tests used? Choose an item.

Quality of Written English

Please indicate the quality of language in the manuscript: Choose an item.

Declaration of Competing Interests

Please complete a declaration of competing interests, considering the following questions:

- Have you in the past five years received reimbursements, fees, funding, or salary from an organisation that may in any way gain or lose financially from the publication of this manuscript, either now or in the future?
- Do you hold any stocks or shares in an organisation that may in any way gain or lose financially from the publication of this manuscript, either now or in the future?
- Do you hold or are you currently applying for any patents relating to the content of the manuscript?
- Have you received reimbursements, fees, funding, or salary from an organization that holds or has applied for patents relating to the content of the manuscript?
- Do you have any other financial competing interests?
- Do you have any non-financial competing interests in relation to this paper?

If you can answer no to all of the above, write 'I declare that I have no competing interests' below. If your reply is yes to any, please give details below.

I have no competing interests.

I agree to the open peer review policy of the journal. I understand that my name will be included on my report to the authors and, if the manuscript is accepted for publication, my named report including any attachments I upload will be posted on the website along with the authors' responses. I agree for my report to be made available under an Open Access Creative Commons CC-BY license (http://creativecommons.org/licenses/by/4.0/). I understand that any comments which I do not wish to be included in my named report can be included as confidential comments to the editors, which will not be published.

Choose an item.

To further support our reviewers, we have joined with Publons, where you can gain additional credit to further highlight your hard work (see: https://publons.com/journal/530/gigascience). On publication of this paper, your review will be automatically added to Publons, you can then choose whether or not to claim your Publons credit. I understand this statement.

Yes Choose an item.
