# Supplementary material for: Profiling the baseline performance and limits of machine learning models for adaptive immune receptor repertoire classification
Source: Gigascience. 2022 May 25;11:giac046. doi: 10.1093/gigascience/giac046 (PMC9154052; doi:10.1093/gigascience/giac046)
Supplement: giac046_Reviewer_2_Report_Original_Submission — Enkelejda Miho -- 9/8/2021 Reviewed [file giac046_reviewer_2_report_original_submission.pdf]

Reviewer Report

Title: Profiling the baseline performance and limits of machine learning models for adaptive immune receptor repertoire classification

Version: Original Submission **Date:** 9/8/2021

Reviewer name: Enkelejda Miho

Reviewer Comments to Author:

General opinion: approved with minor changes
Comments: The manuscript profiles machine learning methods for AIRR T-cell receptor dataset immune state label prediction to establish the baseline performance of such methods across a diverse set of challenges. Simulated datasets with variable properties are used to provide a large amount of benchmarking datasets with known immune state signals while reflecting the natural complexity of experimental datasets. Their results provide insights on the current limits posed by basic dataset properties to baseline ML models and establish a frontier of improvement of AIRR ML research.
The manuscript is understandable and well structured in the approach to comparisons as well as solid conclusions. The graphics are clear and consistent and support the manuscript. Very interesting insight into the importance of single individual variable parameters such as sample size or witness rate on the general accuracy.
The advantage of the results to the scientific community is that it offers an evaluation of classical ML methods, provides large and specialized AIRR benchmark datasets, and allows further development and benchmarking of more sophisticated ML methods.
The manuscript is overall well-written and we endorse it with minor changes:
In paragraph Impact of noise on classification performance (page 14) the sentence "but enriched above a baseline in positive class examples" should be corrected with "but being enriched above a baseline in positive class examples"
In paragraph Machine learning models (methods section, page 21) "lasso" should be corrected with "Lasso".
In paragraph Machine learning models (methods section, page 21) " '- ' " should be corrected with "'-'" and "ð�‘‹jdenotingÂ» with "ð�‘‹j denotingÂ».
In the discussion the sentence "which aligns with the observations that that the majority of the possible contacts between TCR and peptide" should be corrected with "which aligns with the observations that the majority of the possible contacts between TCR and peptide"
Keep comparisons like size>500 and size > 500 concise
Check for missing whitespace as in the description of the figure 1(b): …(5 x 105 % of sequence..
Same in cases like â‰ˆ90% | â‰ˆ 90 % or n=60 | n = 60

Methods

Are the methods appropriate to the aims of the study, are they well described, and are necessary controls included? Choose an item.

Conclusions

Are the conclusions adequately supported by the data shown? Choose an item.

Reporting Standards

Does the manuscript adhere to the journal's guidelines on minimum standards of reporting? Choose an item.

Choose an item.

Statistics

Are you able to assess all statistics in the manuscript, including the appropriateness of statistical tests used? Choose an item.

Quality of Written English

Please indicate the quality of language in the manuscript: Choose an item.

Declaration of Competing Interests

Please complete a declaration of competing interests, considering the following questions:

- Have you in the past five years received reimbursements, fees, funding, or salary from an organisation that may in any way gain or lose financially from the publication of this manuscript, either now or in the future?
- Do you hold any stocks or shares in an organisation that may in any way gain or lose financially from the publication of this manuscript, either now or in the future?
- Do you hold or are you currently applying for any patents relating to the content of the manuscript?
- Have you received reimbursements, fees, funding, or salary from an organization that holds or has applied for patents relating to the content of the manuscript?
- Do you have any other financial competing interests?
- Do you have any non-financial competing interests in relation to this paper?

If you can answer no to all of the above, write 'I declare that I have no competing interests' below. If your reply is yes to any, please give details below.

Enkelejda Miho owns shares is aiNET GmbH.

I agree to the open peer review policy of the journal. I understand that my name will be included on my report to the authors and, if the manuscript is accepted for publication, my named report including any attachments I upload will be posted on the website along with the authors' responses. I agree for my

report to be made available under an Open Access Creative Commons CC-BY license (http://creativecommons.org/licenses/by/4.0/). I understand that any comments which I do not wish to be included in my named report can be included as confidential comments to the editors, which will not be published.

Choose an item.

To further support our reviewers, we have joined with Publons, where you can gain additional credit to further highlight your hard work (see: https://publons.com/journal/530/gigascience). On publication of this paper, your review will be automatically added to Publons, you can then choose whether or not to claim your Publons credit. I understand this statement.

Yes Choose an item.
