# Supplementary material for: Profiling the baseline performance and limits of machine learning models for adaptive immune receptor repertoire classification
Source: Gigascience. 2022 May 25;11:giac046. doi: 10.1093/gigascience/giac046 (PMC9154052; doi:10.1093/gigascience/giac046)
Supplement: giac046_Reviewer_3_Report_Original_Submission — Filippo Castiglione -- 10/6/2021 Reviewed [file giac046_reviewer_3_report_original_submission.pdf]

Reviewer Report

Title: Profiling the baseline performance and limits of machine learning models for adaptive immune receptor repertoire classification

Version: Original Submission **Date:** 10/6/2021

Reviewer name: Filippo Castiglione

Reviewer Comments to Author:

The article "Profiling the baseline performance and limits of machine learning models for adaptive immune receptor repertoire classification by
Kanduri1 et al. describes the construction of suitable reference benchmarks data-sets to guide new AIRR ML classification methods.
The article is interesting and potentially useful in defining benchmark data sets and criteria for constructing specialized AIRR benchmark datasets for the community of researcher interested in AIRR.
The authors following previous indications about model reproducibility and availability also provide a docker container which include all data and procedures to reproduce the study.
The article is sufficiently well written although at time a bit full of details which perhaps could be synthesised further (this has already been done in pictures and tables).
I don't have major concerns. Only a couple of notes.
Would be good to have a figure or diagram showing an example of bags containing receptors and associated witnesses. It could illuminate the reader not familiar with Multiple instanvd learning.
Would be good to have line commands for the generation of data sets (in the case, for instance, of use of Olga). I understand these are inside the docker container but the reader that is not interested in the whole container might find useful to have access to pieces of the pipeline so to use this or that tool (being it in immuneML, in Olga, etc.).
Curiosity: why have the authors used Olga and not the mate Igor?
Why is the performance metric in model training the accuracy and not, for instance, the F1-score? Any particular reason?

Methods

Are the methods appropriate to the aims of the study, are they well described, and are necessary controls included? Choose an item.

Conclusions

Are the conclusions adequately supported by the data shown? Choose an item.

Reporting Standards

Does the manuscript adhere to the journal's guidelines on [minimum standards of reporting?] Choose an item.

Choose an item.

Statistics

Are you able to assess all statistics in the manuscript, including the appropriateness of statistical tests used? Choose an item.

Quality of Written English

Please indicate the quality of language in the manuscript: Choose an item.

Declaration of Competing Interests

Please complete a declaration of competing interests, considering the following questions:

- Have you in the past five years received reimbursements, fees, funding, or salary from an organisation that may in any way gain or lose financially from the publication of this manuscript, either now or in the future?
- Do you hold any stocks or shares in an organisation that may in any way gain or lose financially from the publication of this manuscript, either now or in the future?
- Do you hold or are you currently applying for any patents relating to the content of the manuscript?
- Have you received reimbursements, fees, funding, or salary from an organization that holds or has applied for patents relating to the content of the manuscript?
- Do you have any other financial competing interests?
- Do you have any non-financial competing interests in relation to this paper?

If you can answer no to all of the above, write 'I declare that I have no competing interests' below. If your reply is yes to any, please give details below.

I declare that I have no competing interests

I agree to the open peer review policy of the journal. I understand that my name will be included on my report to the authors and, if the manuscript is accepted for publication, my named report including any attachments I upload will be posted on the website along with the authors' responses. I agree for my report to be made available under an Open Access Creative Commons CC-BY license (http://creativecommons.org/licenses/by/4.0/). I understand that any comments which I do not wish to be included in my named report can be included as confidential comments to the editors, which will not be published.

Choose an item.

To further support our reviewers, we have joined with Publons, where you can gain additional credit to further highlight your hard work (see: https://publons.com/journal/530/gigascience). On publication of this paper, your review will be automatically added to Publons, you can then choose whether or not to claim your Publons credit. I understand this statement.

Yes Choose an item.
